# The role of NOI-domain containing proteins in plant immune signaling

**DOI:** 10.1186/1471-2164-14-327

**Published:** 2013-05-14

**Authors:** Ahmed J Afzal, Jin Hee Kim, David Mackey

**Affiliations:** 1 Department of Horticulture and Crop Science, The Ohio State University, Columbus, OH, USA; 2 Department of Molecular Genetics, The Ohio State University, Columbus, OH, USA

## Abstract

Here we present an overview of our existing knowledge on the function of RIN4 as a regulator of plant defense and as a guardee of multiple plant R-proteins. Domain analysis of RIN4 reveals two NOI domains. The NOI domain was originally identified in a screen for nitrate induced genes. The domain is comprised of approximately 30 amino acids and contains 2 conserved motifs (PXFGXW and Y/FTXXF). The NOI gene family contains members exclusively from the plant lineage as far back as moss. In addition to the conserved NOI domain, members within the family also contain conserved C-terminal cysteine residue(s) which are sites for acylation and membrane tethering. Other than these two characteristic features, the sequence of the family of NOI-containing proteins is diverse and, with the exception of RIN4, their functions are not known. Recently published interactome data showing interactions between RIN4 and components of the exocyst complex prompt us to raise the hypothesis that RIN4 might be involved in defense associated vesicle trafficking.

## Introduction

Microbes induce resistance responses in plants by activating two distinct branches of the plant immune system [1,2]. PTI (PAMP-triggered immunity) is activated upon recognition of pathogen associated molecular patterns (PAMPs), which are ubiquitous bacterial features such as epitopes within the bacterial flagellin protein [3,4]. PAMPs are recognized by pattern recognition receptors (PRR) and comprise the first layer of the plant defense response. Pathogens respond by delivering PTI-suppressing virulence factors, *e.g.* effector proteins injected from Gram-negative bacteria into the cytosol of plant cells through the needle-like Type III secretion system (TTSS). Furthering the arms race, plant-encoded resistance (R) proteins directly or indirectly recognize virulence effectors and activate effector-triggered immunity (ETI).

### R-protein activation – direct or indirect

R-proteins are sometimes activated dependent on direct interaction with their cognate effector protein(s) [1]. For example, Y2H screens have showed that (1) the Pi-ta R-protein of rice interacts with its cognate AvrPita effector from *Magnaporthe grisea*[5], (2) the L and M R-proteins from flax interact with their cognate AvrL567 and AvrM effectors from flax rust [6], and (3) the RRS1-R R-protein of *Arabidopsis thaliana* interacts with its cognate PopP2 effector from *Ralstonia solanacearum*[7]. However, the lack of evidence of direct interaction for numerous other R-protein/cognate effector pairs led to formulation of the guard hypothesis, which states that R-proteins can indirectly recognize pathogen effectors by sensing perturbations they induce. The plant protein (or other type of molecule), of which a perturbed state is recognized by the R-protein, is known as the “guardee”. Guardees can be either direct or indirect virulence targets of effectors or target decoys [8,9].

Numerous studies over the last decade have lent support to the guard hypothesis. The *Arabidopsis thaliana* R-protein RPS5 is activated when PBS1 (serine threonine kinase), is cleaved by the *Pseudomonas syringae* cysteine protease effector protein, AvrPphB [10,11]. Another guardee protein, RIN4, is modified by at least four effectors from *Pseudomonas syringae*. HopF2, a T3E with ADP-ribosyltransferase activity targets RPM1-Interacting Protein 4 (RIN4) [12,13]. AvrB and AvrRpm1 induce phosphorylation of RIN4 [14-16] and AvrRpt2, a cysteine protease, cleaves RIN4 at two cleavage sites (RCS1 and RCS2) [17-19]. AvrB-, AvrRpm1-, and AvrRpt2-induced perturbations of RIN4 lead to the induction of ETI by at least two *Arabidopsis thaliana* R-proteins, RPM1 and RPS2 [14,15,20-23]. Whether HopF2-induced modification of RIN4 can also elicit R-protein mediated defense is not known.

R-proteins that “guard” RIN4 arose independently in a variety of plant species. RIN4 homologs regulate innate immunity in tomato [24] lettuce [25] and soybean [26]. Soybean contains 4 homologs of the RIN4 protein (GmRIN4a-GmRIN4d). Amino acid alignments show that GmRIN4a and GmRIN4b are 50% identical to AtRIN4 whereas GmRIN4c and GmRIN4d are 46% and 48% identical to AtRIN4 respectively [26]. Resistance to AvrB and AvrRpm1 in soybean is mediated by the R-proteins RPG1-b and RPG1-r, respectively. Although functionally similar, RPG1-b and RPM1 are evolutionarily distinct [27]. Additional resistance genes specific to AvrRpm1, only one of which originates in the Rpg locus, have arisen in common bean [28]. In lettuce hybrids, necrotic lesions result from an allelic interaction between RIN4 (54% identical to AtRIN4) and a second locus, with both loci contributing to quantitative resistance against a virulent race of *Bremia lactucae* and race specific resistance to an avirulent race of the same pathogen. It is tempting to speculate that the second locus that interacts with RIN4 is an R-gene. In any case, the independent evolution of R-proteins that guard RIN4 highlights the significance of RIN4 to plant defense.

Here we will describe in greater detail what is known about the function of RIN4 as a regulator of plant defense and as a guardee of multiple plant R-proteins.

### Sequence / structure analysis of RIN4 protein

A lack of discernible structural features in RIN4 makes prediction of function difficult. Although domain analysis of RIN4 reveals two NOI domains (pfam05627), little is known about their role in plant defense. Proteins containing NOI domains were initially identified in a screen for nitrate-induced genes (NOI stands for NO_3_-Induced). No link between NOI proteins and nitrogen metabolism has been established. The family of proteins containing NOI domains contains members exclusively from the plant lineage as far back as moss. In addition to the conserved NOI domain, family members also contain conserved C-terminal cysteine residue(s). Other than these two characteristic features, NOI-containing proteins share no apparent homology and, with the exception of RIN4, their functions are not known.

RIN4 has two NOI domains located at the N- and C-terminal ends of the protein (termed N-NOI and C-NOI, respectively). We previously conducted phylogenetic analysis to examine the relation of N-NOI and C-NOI protein domains from RIN4 and its most closely related homologs from a variety of other monocots and dicots, as well as, moss. Even though the proteins came from significantly diverged plants, the N-NOI and C-NOI domains grouped into separate clades, indicating that they have evolved independently from one another [29]. Consistent with this interpretation are observations that the N-NOI and C-NOI of RIN4 function distinctly; the C-NOI interacts with AvrB and is required for RPM1-mediated ETI [15,30].

There are two conserved motifs within the NOI domains. The first conserved motif (PXFGXW) is the RIN4-cleavage site (RCS) targeted by AvrRpt2 [18]. With the exception of three RIN4 homologs from *Arabidopsis thaliana* (At5G48500, At5G48657 and At3G07195), all examined homologs contain an RCS in the C-terminal NOI domain. Cleavage of RCS2 by AvrRpt2 is critical for the activation of RPS2 [19,20]. The second conserved motif is the sequence Y/FTXXF, which is highly conserved in the center of the NOI domain.

The type III effectors AvrRpm1 and AvrB induce phosphorylation of the conserved threonine within the Y/FTXXF motif of both the N-NOI and C-NOI of *Arabidopsis thaliana* RIN4. Notably, threonine phosphorylation within the C-NOI is key to the activation of ETI by RPM1 [14,15]. Since effectors target specifically the two conserved motifs within the NOI domains of RIN4, they likely also target these same motifs within other NOI-containing proteins. In fact, in addition to RIN4, several other NOI-containing proteins from *Arabidopsis thaliana* were shown to be cleaved by AvrRpt2 [18,31]. Thus, it is a reasonable hypothesis that the NOI-family of proteins control plant processes, most likely biotic defense, that bacterial pathogens aim to perturb. The conservation of the RCS and Y/FTXXF motifs targeted by T3Es within the NOI domains likely results from the evolutionarily more ancient function of these motifs as targets for cellular proteins. Indeed, the phosphorylation of *Arabidopsis thaliana* RIN4 is mediated by an *Arabidopsis thaliana* receptor like protein kinase (RIPK) and, in tomato and tobacco, an endogenous protease has been shown to cleave the RCS [14,24].

**Figure 1:**
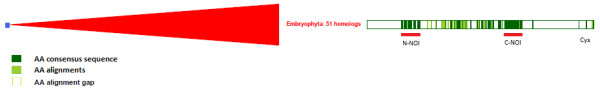
**The protein alignment composite of 51 NOI containing proteins from monocots, dicots and moss was generated using the Ensembl plant database (**http://plants.ensembl.org/**).** The 51 NOI containing proteins were selected on the basis of their sequence homology to *At*RIN4. Green bars show areas of amino acid alignment while the white areas are gaps in the alignment. Dark green bars indicate the consensus alignment in the collapsed tree. The regions corresponding to the C- and N- NOI domains are shown by red bars. The highest level of conservation in sequence is seen in the C-NOI domain, followed by the N-NOI domain and the region representing the conserved C-terminal cysteines.

**Figure 2:**
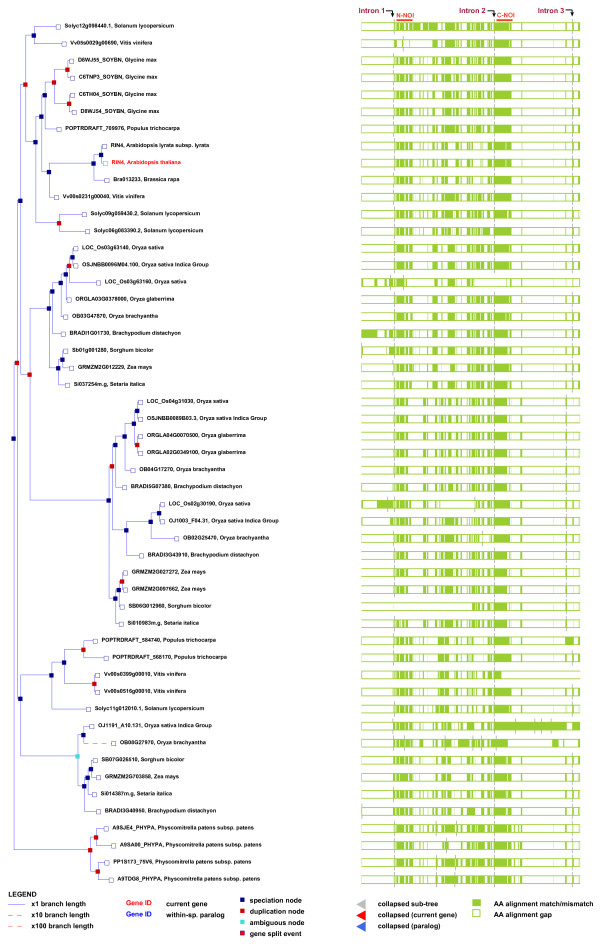
**(Left panel) A Phylogenetic tree of 51 NOI containing proteins from monocots, dicots and moss was generated using the Ensembl plant database *(***http://plants.ensembl.org/***).*** The 51 NOI containing proteins were selected on the basis of their sequence homology to *At*RIN4. Red nodes in the phylogenetic tree represent duplication events whereas the blue nodes are speciation events. (Right panel) Multiple alignments of the 51 NOI containing proteins shown on the left were generated using MUSCLE. The light green bars correspond to amino acids within the protein sequence, whereas the white spaces represent alignment gaps. The 3 intron junctions are shown as black lines (labeled) that run perpendicular to the alignment trace. The intron junction preceding the N-NOI is labeled as intron 1 whereas the intron junction preceding the C-NOI is labeled as intron 2. The position of the third intron varies between monocots and dicots. This junction in most monocots and moss is one amino acid removed from the conserved cysteines, whereas in most dicots it immediately precedes the C-terminal cysteine residues. The regions corresponding to the N-NOI and C-NOI are shown by red bars.

For our current analysis we generated a phylogenetic tree composed of 51 RIN4 homologs, each containing two NOI domains, from monocots, dicots and moss. The tree was generated using the entirely automated plant Ensembl database (http://plants.ensembl.org). The alignment composite generated from 51 RIN4 homologs shows greater sequence conservation and reduced gaps in the C-NOI in comparison to the N-NOI (Figure 1). We also analyzed the position of the exon junctions relative to the full-length protein. Most of the analyzed NOI proteins contain 3 introns. The intron that precedes the C-NOI (Intron 2) is conserved in all but 3 (Os03g63160, OB02G25470 and OB08G27970) sequences (Figure 2), while the intron preceding the N-NOI is conserved in 41 of the 51 analyzed proteins. Collectively, these data indicate that the C-NOI may be the ancestral version and most well conserved, while the N-NOI may have arisen through independent duplications that only sometimes retained the intron and are better able to diverge in amino acid sequence (Figure 2). The position of the third intron varies between monocots and dicots. This junction in most monocots and moss is one amino acid removed from the conserved cysteines, whereas in most dicots it immediately precedes the C-terminal cysteine residues (Figure 2). All analyzed RIN4 homologs from moss have additional introns between the two conserved introns. The most parsimonious explanation for exon evolution in angiosperms is that the additional junctions were lost after the split between moss and vascular plants. Alternatively, it is possible that the extra introns were lacking in the moss-vascular plant common ancestor and were acquired after divergence of moss.

We also generated alignments of 13 RIN4-like *Arabidopsis* proteins, selected on the basis of the plant-specific NOI domains and the conserved C terminal cysteine residues (Additional file 1: Figure S1). C-terminal sequences are important for sub-cellular localization of RIN4. With few exceptions, the NOI-containing RIN4 homologs (Figure 1), as well as other NOI containing proteins from *Arabidopsis thaliana* (Additional file 1: Figure S1), contain one to three cysteines within the C-terminal twelve amino acids that, except for in moss RIN4, are closely flanked by one or more aromatic residues [29]. In *Arabidopsis thaliana* RIN4, the C-terminal cysteines and the phenylalanine residues that follow are required for acylation and plasma membrane attachment [32]. Given the conservation of these residues at their C-termini, acylation and membrane association is likely a common feature of NOI-containing proteins and may relate to their function. Consistent with this prediction, the C-terminal cysteines of RIN4 are required for supporting the function of RPM1 and negatively regulating the function of RPS2 (Luis da Cunha and D.M., unpublished data and [20]). Also, RIN4 derivatives lacking the C-terminal cysteines are hyperactive suppressors of PTI [29]. Thus, membrane tethering of RIN4 affects, both positively and negatively, its defense regulating functions in *Arabidopsis thaliana*. In *Arabidopsis* other than RIN4 only AT3G07195 and AT5G48657 contain both N- and C- terminal NOI domains. 3 of the examined NOI containing proteins (At5G48657, At5G48500 and At3G07195) lack the predictive AvrRpt2 cleavage site in the C-NOI domain. However these 3 proteins contain the conserved AvrRpt2 cleavage site (RCS motif) within the first 20 residues of the N-terminus (Additional file 1: Figure S1). On the basis of the conservation of the AvrRpt2 cleavage site in the NOI-family of proteins, we can speculate that bacterial effectors aim to perturb a process important for biotic defense. Additionally, the conservation of the RCS motif targeted by T3Es within the NOI may allow NOI- containing proteins to function as decoys [9].

### Role of NOI domains in suppression of PTI

RIN4 was previously shown to negatively regulate PTI [33]. Over-expression of RIN4 suppressed PTI and PTI responses were enhanced in the absence of RIN4. To measure the relative contribution of the N- and C-NOI domains in PTI regulation, the ability of over-expressed RIN4 derivatives to suppress flg22-induced callose deposition (flg22 is a PAMP from the bacterial flagellin protein) and enhance the growth of TTSS-deficient bacteria was determined [29]. A derivative of RIN4 lacking both NOI domains was unable to suppress PTI while derivatives lacking either NOI alone maintained the ability to suppress PTI. It therefore seems that the evolutionarily distinct N-NOI and C-NOI can each contribute to PTI suppression by over-expressed RIN4. However, this data does not exclude the possibility that distinct functions of each NOI domain would be detected under different experimental conditions or with different readouts for PTI.

AvrRpm1, AvrB, and AvrRpt2 can each perturb RIN4 and inhibit PTI [33,34]. Thus, it has been speculated that targeting of RIN4 contributes to the virulence function of these T3Es. However, in the case of AvrRpt2, it seemed counter-intuitive that this effector would cleave a negative regulator of PTI. A number of potential explanations have been provided for this observation. First, one or more NOI-containing proteins targeted by AvrRpt2 [18] may be bonafide virulence target(s) that regulate PTI. Consistent with this hypothesis is the observation that AvrRt2 is able to promote virulence in plants lacking RIN4 [27] presumably by targeting other NOI contacting proteins. Indeed, a recent study showed that inducible expression of AvrRpt2 decreases the level of three NOI-containing proteins in plasma membrane preparations of *Arabidopsis thaliana* (NOI4, NOI6 and NOI7) [31]. In this scenario, RIN4 might only serve as a decoy rather than as a bonafide virulence target of AvrRpt2 [9]. A second possibility is that degradation of RIN4 disrupts the function of associated proteins. For example, RIN4 interacts in guard cells with autoinhibited H^+^ ATPases (AHA proteins) that promote the opening of stomata [35]. Stomatal closure is a PTI response that restricts bacterial invasion from the leaf surface [36]. H^+^ ATPase activity is reduced in *rin4* mutant plants, which tends to keep stomata closed and thus enhances this PTI response. However, disrupting the function of AHA proteins by elimination of RIN4 would enhance defense, contrary to the virulence function of AvrRpt2. Thus, in this scenario, elimination of RIN4 by AvrRpt2 would need to disrupt the function of another protein(s) that promote, rather than suppress, plant defense. Putative RIN4-interacting proteins involved in vesicle secretion, discussed below, may fit these criteria. Our recent findings support a third possibility that cleavage of RIN4 by AvrRpt2 does not simply eliminate RIN4, but rather produces fragments that effectively suppress PTI [29]. Consistent with this third idea, we have shown that the Avrpt2-cleavage products remain detectable *in planta* after cleavage of RIN4 by bacterially delivered AvrRpt2. Furthermore, the non-membrane localized ACP2 (AvrRpt2 Cleavage Product 2) and the membrane localized ACP3 (AvrRpt2 Cleavage Product 3) fragments are hyperactive PTI suppressors. ACP2, which is the fragment bounded by RCS1 and RCS2, contains most of the N-NOI and ACP3, which is the fragment C-terminal to RCS2, contains most of the C-NOI. According to the decoy model, a decoy functions by mimicking the actual effector target and should have no affect on pathogen fitness when the cognate R protein is not activated [9]. Hence our data support the hypothesis that RIN4 is a bonafide virulence target of AvrRpt2 and indicates that cleavage of RIN4 by AvrRpt2 may activate its ability to suppress PTI.

We hypothesize that other effectors target RIN4 to enhance its defense suppressing activity. In soybean, GmRIN4 proteins regulate basal defense and may be virulence targets of AvrB and AvrRpm1 [26,37,38]. AvrB and AvrRpm1 induce phosporylation of *Arabidopsis thaliana* RIN4 at the conserved threonines within the N-NOI and C-NOI [14,15]. It is therefore tempting to speculate that NOI phosphorylation suppresses plant defense. The ability of AvrB and AvrRpm1 to suppress PTI responses in plants lacking RIN4 indicates that RIN4 is not their only virulence target [33,39]. Defense suppression may also be mediated through targeting of the conserved threonine in other defense-regulating NOI-containing proteins. The action of HopF2 can suppress plant defense in a manner that requires RIN4, perhaps via ADP-ribosylation of RIN4 [12,13]. Cellular proteins that target these motifs within the NOI, *e.g.* plant proteins that cleave RCS sites or phosphorylate the conserved threonine [14,24], may function as defense regulators. In this scenario, the effectors that suppress defense by modifying RIN4 and other NOI proteins have co-opted a cellular defense-regulatory process.

### Role of NOI domains in activation of ETI

Our RIN4 structural model [29] predicts that the two NOIs interact extensively. This could help explain the observed intermolecular interactions between RIN4 homologs in soybean, which may be required for function of the Rpg1-b R-protein [26]. Structural changes and sub-cellular relocation brought about by T3E perturbations could activate R-proteins by promoting or disrupting intra- or inter-molecular NOI:NOI interactions and/or interactions of RIN4 with heterologous protein partners.

In *Arabidopsis thaliana*, RPM1 and RPS2 are predicted to be cytosolic, but localize to the plasma membrane [21,35,40]. RIN4 prevents ectopic activation of RPM1 and RPS2, perhaps via their interaction at the plasma membrane [16,20-22,39]. The C-NOI domain of RIN4 can complement AvrB and AvrRpm1 triggered RPM1 function whereas N-NOI is unable to do so. Both effectors associate with and induce phosphorylation of RIN4 although neither has known kinase activity [16]. Recently RIPK has been identified as the kinase that phosphorylates RIN4 at three residues (T21, S160 and T166) in both NOI domains [14]. Phospho-mimic substitutions at T166 in the C-NOI caused effector-independent RPM1 activation. However, T166 is not essential for AvrRpm1 dependent HR in *N. Benthamiana* but is required for AvrB mediated RPM1 activation in *Arabidopsis*[15]. AvrRpm1- or AvrB-induced phosphorylation does not release RIN4 from the plasma membrane and leads to the activation of RPM1 at the plasma membrane [41]. Phosphorylated RIN4 may activate RPM1 by recruiting proteins required for R-protein mediated signaling or alleviate its negative regulation of RPM1. A residue analogous to T166 is conserved in all NOI containing proteins, hence this residue may function beyond the regulation of RPM1 [15].

The subcellular location of RPS2 activation is unknown. The ectopic activation of RPS2 in *rin4* mutant plants differs genetically from AvrRpt2-induced activation of RPS2. RPS2-induced HR in response to AvrRpt2 is non-race-specific disease resistance 1 (NDR1)-dependent [28] whereas the ectopic activation of RPS2 in plants lacking *rin4* is NDR1-independent [39]. Interestingly, ACP3 of RIN4 has been shown to interact with plasma membrane localized NDR1 [42]. Thus, activation of RPS2 by AvrRpt2 may require signaling steps including NDR1:ACP3 interaction and a novel function for ACP2 that could include release from the plasma membrane and movement to a new sub-cellular location.

### Putative RIN4-interacting proteins

To determine additional targets of RIN4 and other *Arabidopsis thaliana* NOI containing proteins, we searched the Arabidopsis Interactome Mapping database (AI-1) and the plant-pathogen immune network database (PPIN-1) [43,44]. PPIN-1 contains 3148 interactions, 843 *Arabidopsis thaliana* immune related proteins and 83 combined effectors from *Pseudomonas syringae* and *Hyaloperonospora arabidopsidis* whereas the AI-1 network contains 6205 interactions and 2,774 *Arabidopsis thaliana* proteins. We found 9 interacting partners for RIN4 using the PPIN-1 database (Table 1). The known RIN4 interactors RPM1 [16] and RPS2 [21] were missed in the screen, although for RPS2 it is not clear if the interaction is direct or indirect. Other known RIN4 interactors, AvrB and NDR1, were detected (Table 1). An uncharacterized CC-NB-LRR protein (AT1G12290) was also found to interact with RIN4, raising the possibility of RIN4 being guarded by additional R-proteins. From PPIN-1, we found interactors for 2 NOI containing proteins. We also found interactors for 2 of the 10 *Arabidopsis thaliana* NOI-containing proteins represented in AI-1. NOI6 and RIN4, each interact with proteins belonging to the Cys/His-rich and Exo70 protein families. Thus, interactions with these proteins likely are mediated through the NOI domain. The AI-1 and PPIN-1 databases contain only a subset of the entire immune repertoire, thus many NOI-R interactions were not detected. However, the existence of 15 NOI-containing proteins in *Arabidopsis thaliana* and of similarly large gene families in other sequenced plants indicates that they play generally important roles in plants, including perhaps plant defense, and therefore may be guarded by R-proteins.

**Table 1:** Interactors of RIN4 and NOI containing proteins in the AI-1 and PPIN-1 databases

**Accession**	**Interactors (PPIN-1)**	**Interactors (AI-1)**
**RIN4 (AT3G25070)**	Unknown (AT3G01670)	
	Cysteine/Histidine-rich (AT3G46810)	
	EXO70E2 (AT5G61010)	
	EXO70B1 (AT5G58430)	
	NDR1 (AT3G20600)	
	NB-LRR (AT1G12290)	
	Cysteine/Histidine-rich (AT2G19650)	
	bZIP transcription factor (AT3G51960)	
	Pseudomonas syringae effector AvrB	
**NOI9 (AT5G48500)**	Anaphase-promoting complex (AT3G48150) Unknown (AT4G01090)	Anaphase-promoting complex (AT3G48150)
**NOI3 (AT2G17660)**	HSP81-3 (AT5G56010)	
**N0I6 (AT5G64850)**		Cysteine/Histidine-rich (AT4G01920)
		EXO70A1 (AT5G03540)

Targets of RIN4 and other *Arabidopsis thaliana* NOI containing proteins were determined using the Arabidopsis Interactome Mapping database (AI-1) and the plant-pathogen immune network database (PPIN-1). The PPIN-1 contains 3148 interactions, 843 *Arabidopsis* immune related proteins and 83 combined effectors from *Pseudomonas syringae* and *Hyaloperonospora arabidopsidis* whereas the AI-1 network contains 6205 interactions and 2,774 *Arabidopsis* proteins. From PPIN-1, we found interactors for 3 NOI containing proteins whereas the AI-I database search resulted in interactors for 2 NOI-containing proteins.

The evolutionarily most ancient function of RIN4 may have been in the regulation of PTI, specifically limiting the detrimental effects of inappropriate defense signaling. As a defense regulator, it may have been a ripe target for bacterial effector proteins, and consequently for subsequent guarding by R-proteins. R-protein activation emanating from perturbation of RIN4 might involve cellular reprogramming that restores the signaling pathway originally targeted by T3Es. Supporting this idea are observations that numerous genes are required for both PTI- and ETI-signaling, *e.g.* phytoalexin deficient 4 (PAD4) and NDR1 [45] and that there is significant overlap in the immune responses, such as the expression of defense related genes and callose deposition, during PTI and ETI [46,47]. More generally, ETI may be envisioned as the protection/restoration and amplification of PTI responses. Thus, proteins that interact with RIN4 in a biologically significant manner might have roles in PTI and/or ETI.

### A putative role for RIN4 in regulating defense-associated vesicle trafficking

An ADP ribosylation factor-guanine nucleotide exchange factor (ARF-GEF) protein HopM1 interacting protein 7 (AtMIN7/BIG5) is required for elicitation of both PTI and ETI in *Arabidopsis thaliana*. The protein is a vesicle traffic regulator in *Arabidopsis thaliana* which is destabilized by HopM1, a type III effector protein from *P. syringae*[48-50]. It is therefore likely that one mode of PTI suppression by bacterial effectors is mediated by an ability to interfere with the defense-associated vesicle trafficking pathway*.* Activation of AvrRpt2-induced ETI results in the stabilization of AtMIN7 in transgenic plants expressing HopMI, thereby reinforcing the PTI response [48]. AvrRpt2-mediated RPS2 activation also induces the activation of additional proteins involved in vesicular trafficking [31]. Thus, AvrRpt2-induced perturbation of RIN4 and the subsequent activation of RPS2 can be envisioned as a restoration of the vesicular trafficking network necessary for PTI. Our previous studies failed to show an effect of RIN4 over-expression on early signaling events during PTI [29]. Moreover, the PPIN-1 and AI-1 interactors for RIN4 and NOI6 include EXO70 proteins (Table 1). Interactions between RIN4 and a component of the exocyst complex, which regulates vesicle trafficking, prompts us to raise the hypothesis that RIN4 might be involved in defense-associated vesicle trafficking. RIN4 may guide or activate the polarized secretion of defense-related vesicles toward the bacterial infection site. Alternatively, EX070 complexes might recognize newly synthesized RIN4 proteins, driving RIN4 movement toward plasma membranes under pathogen attack.

The plant immune system uses secretory vesicles to transfer defense-related cargoes toward the plasma membrane at the site of pathogen invasion [51]. Several studies have suggested that the secretory system is one of the crucial factors in the battle between pathogen attack and host defense. Pathogen effectors inhibit host trafficking. The suite of *P. syringe* type III effectors preferentially repress a set of *Arabidopsis thaliana* genes encoding secreted proteins and disrupt expression of genes involved in the secretion processes [52,53]. *X. campestris pv. vesicatoria* effector protein XopJ inhibits apoplastic accumulation of a secreted version of GFP [54]. HopZ1a expressed by some strains of *P. syringae* disrupts the microtubule network and disrupts secretion [55]. On the other hand, host plants have adapted to increase capacity of the secretory system during induced defense. SA-dependent activation of non-expressor of pathogenesis related 1 (NPR1) regulates the induction of *PR*-genes, the products of which are secreted proteins, as well as a large set of genes encoding proteins involved in facilitating secretion [56]. Expression of exocyst components including EXO70B2, H1, H2, and H7 is responsive to treatment of elicitor proteins [53,57,58]. Mutations of *Exo70B1* or *Exo70H2* enhance susceptibility to fungal and bacterial infection [58].

## Conclusions

In the last decade, significant progress has been made towards examining the role of RIN4 as a plant defense regulator. Interaction of RIN4 with the exocyst complex prompts us to raise the hypothesis that RIN4 might be involved in defense associated vesicle trafficking. Although the importance of vesicle-mediated transport in plant immunity is well established, knowledge of the contents of defense-associated vesicles is limited. Also, the regulatory mechanisms that connect recognition of pathogen to enhanced vesicle transport and the molecular mechanisms by which vesicle trafficking is polarized toward the site of infection need better resolved. The role of RIN4 in one or more of these processes remains to be determined.

## Abbreviations

NOI: Nitrate induced; PAMP: Pathogen-associated molecular patters; ETI: Effector-triggered immunity; PTI: PAMP-triggered immunity; TTSS: Type III secretion system; RIN4: RPM1 interacting protein 4; RCS: RIN4 cleavage site; PPIN: Plant-pathogen immune network; NDR1: Non-race-specific disease resistance 1; ARF-GEF: ADP ribosylation factor-guanine nucleotide exchange factor; PAD4: Phytoalexin deficient 4; CC-NB-LRR: Coiled coil*-*nucleotide binding*-*leucine rich repeat; ACP: AvrRpt2-cleavage product; PR1: Pathogenesis related 1; NPR1: Non-expressor of pathogenesis related 1.

## Competing interests

Authors declare that they have no competing interests.

## Authors’ contributions

DM and AJA conceived of the study. AJA carried out the NOI interactome analysis and generated the phylogenetic tree and protein alignments. DM, AJA and JHK wrote the manuscript. AJA and DM edited the final version. All authors read and approved the final manuscript.

## Supplementary Material

Additional file 1: Figure S1 ClustalW alignment of RIN4 and 13 *Arabidopsis* NOI containing proteins (AT5G48657, AT5G48500, AT3G07195, AT5G19473, AT5G09960, AT5G64850, AT5G55850, AT2G04410, AT5G63270, AT3G48450, AT5G40645, AT2G17660 and AT4G35655) was performed on the EBI server. The C-NOI domain is outlined in orange whereas the C-terminal cysteine residues are highlighted by the black box. The AvrRpt2 cleavage site (red arrow) lies within the consensus PxFGxW motif (red box). In addition to RIN4 only AT3G07195 and AT5G48657 contain both N- and C-NOI domains. 3 out of the 14 proteins (At5G48657, At5G48500 and At3G07195) lack the predictive AvrRpt2 cleavage site in the C-NOI domain. However the 3 proteins contain the conserved AvrRpt2 cleavage site within 20 amino acids of the N-terminus. All the analyzed proteins contain 1–3 C-terminal cysteine residues required for palmitoylation and membrane attachment found in RIN4. The alignments were edited and visualized using Jalview. Click here for file
