# Supplementary figures and images for: The role of NOI-domain containing proteins in plant immune signaling
Source: BMC Genomics. 2013 May 14;14:327. doi: 10.1186/1471-2164-14-327 (PMC3661340; doi:10.1186/1471-2164-14-327)

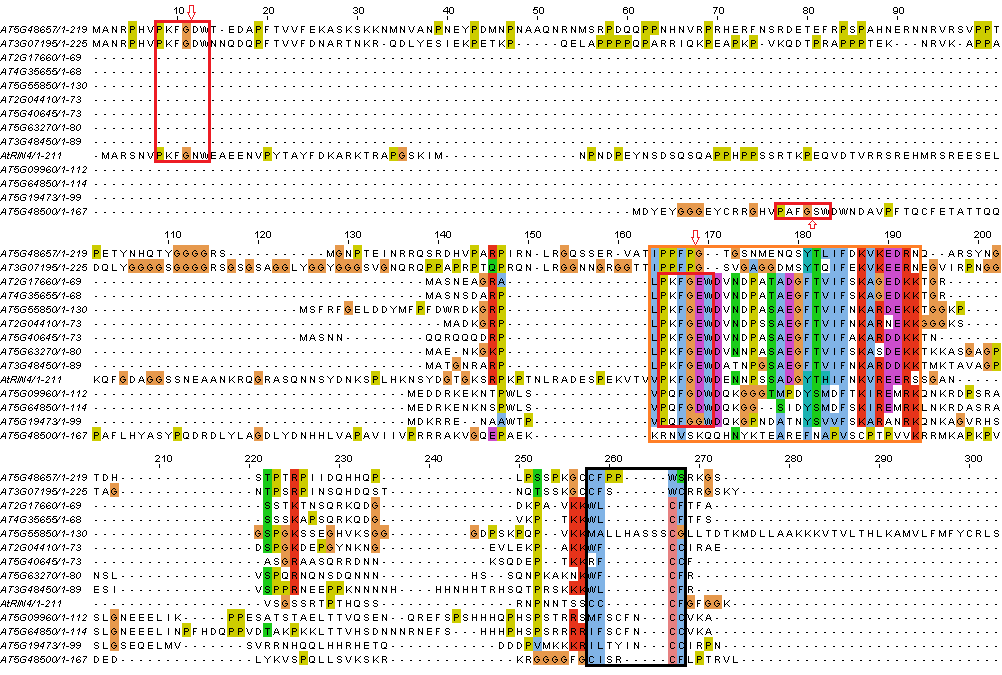

Supplement: Additional file 1: Figure S1 — ClustalW alignment of RIN4 and 13 Arabidopsis NOI containing proteins (AT5G48657, AT5G48500, AT3G07195, AT5G19473, AT5G09960, AT5G64850, AT5G55850, AT2G04410, AT5G63270, AT3G48450, AT5G40645, AT2G17660 and AT4G35655) was performed on the EBI server. The C-NOI domain is outlined in orange whereas the C-terminal cysteine residues are highlighted by the black box. The AvrRpt2 cleavage site (red arrow) lies within the consensus PxFGxW motif (red box). In addition to RIN4 only AT3G07195 and AT5G48657 contain both N- and C-NOI domains. 3 out of the 14 proteins (At5G48657, At5G48500 and At3G07195) lack the predictive AvrRpt2 cleavage site in the C-NOI domain. However the 3 proteins contain the conserved AvrRpt2 cleavage site within 20 amino acids of the N-terminus. All the analyzed proteins contain 1–3 C-terminal cysteine residues required for palmitoylation and membrane attachment found in RIN4. The alignments were edited and visualized using Jalview. [file 1471-2164-14-327-S1.tiff]
